# Temperature and time dependence on extraction of Molybdenum-99 hot atoms from neutron-irradiated β-molybdenum trioxide particles into water†

**DOI:** 10.1039/d5ra01952d

**Published:** 2025-05-22

**Authors:** Ying Yang, Minh Chu Ngo, Taiga Kitagawa, Yoshitaka Fujita, Yukiko Takahashi, Tatsuya Suzuki, Tadachika Nakayama, Thi Mai Dung Do, Koichi Niihara, Hisayuki Suematsu

**Affiliations:** a Extreme Energy-Density Research Institute, Nagaoka University of Technology Nagaoka Niigata 940-2188 Japan; b Department of Japan Materials Testing Reactor, Japan Atomic Energy Agency Oarai Ibaraki 311-1393 Japan; c Department of Materials Science and Engineering/Bioengineering, Nagaoka University of Technology Nagaoka Niigata 940-2188 Japan; d Department of Nuclear Technology, Nagaoka University of Technology Nagaoka Niigata 940-2188 Japan s205074@stn.nagaokaut.ac.jp

## Abstract

This study presents a novel perspective by examining the influence of temperature and heating time on the extraction of ^99^Mo into water, contributing to a better understanding of its extraction kinetics. β-MoO_3_ particles were synthesized *via* thermal evaporation and subsequently characterized using X-ray diffraction and transmission electron microscopy. These particles were then neutron-irradiated at the Kyoto University Research Reactor facility, after which the activity of the ^99^Mo in the particles was analyzed by a high-purity germanium semiconductor detector. The irradiated β-MoO_3_ particles were dispersed in water at temperatures of 20, 30, 40 or 50 °C for durations of 1, 2.5 or 5.5 h to extract ^99^Mo. The associated β-MoO_3_ solutions were also analyzed by a high-purity germanium semiconductor detector. The ^99^Mo extraction efficiency was increased from 20.31 ± 1.24% to 66.88 ± 1.42% upon raising the water temperature and increasing the heating duration. The activation energy for this extraction process was found to be lower than that for simple atomic diffusion in crystalline MoO_3_ and higher or close to that for the formation of or proton conduction in a hydrated MoO_3_ phase. This result suggests that a hydrated MoO_3_ phase accelerated the extraction of ^99^Mo. To the best of our knowledge, this is the first research to investigate the temperature and time dependence in the extraction of ^99^Mo hot atoms, providing a promising approach for large-scale production of ^99^Mo/^99m^Tc radiopharmaceuticals.

## Abstract 2

The new extraction process significantly improves the extraction of Molybdenum-99 hot atoms/Tc-99m

## Graphical abstract

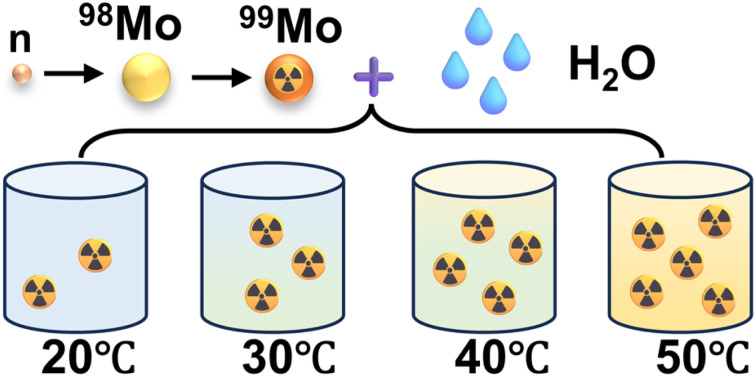

## 1. Introduction

The technetium-99m (^99m^Tc) radioisotope is commonly used in medical applications.^1,2^ Specifically, this isotope is employed for imaging of various anatomical structures including bones, the myocardium, the cerebral cortex, the thyroid gland and the pulmonary system, as well as for numerous specialized medical examinations.^3–6^ The use of ^99m^Tc is important in various nuclear diagnostic procedures for two primary reasons. Firstly, the short half-life of this isotope reduces the radiation dose absorbed by the patient.^7^ Secondly, this element emits gamma photons at 140 eV with an abundance of 89%, which is highly advantageous with regard to imaging.^8^ Molybdenum-99 (^99^Mo) is the parent nuclide of ^99m^Tc^9^ and is typically produced *via* the ^98^Mo (n, γ) ^99^Mo or (n, f) reactions.^10,11^ However, due to the shutdown of nuclear reactors with highly enriched uranium fuel in many countries, the ability to use the (n, f) reaction process to obtain ^99m^Tc is currently limited.^12^ In contrast, the ^98^Mo (n, γ) ^99^Mo reaction not only produces minimal amounts of radioactive waste, but also fully complies with the Nuclear Non-Proliferation Treaty^13,14^ and so has been selected for long-term development.

The Japan Atomic Energy Agency (JAEA) has been engaged in the development of techniques for the generation of ^99m^Tc from ^99^Mo through the (n, γ) reaction since 2010.^15^ Unfortunately, the specific activity of ^99m^Tc radiopharmaceuticals produced by this method is lower than that achieved using the (n, f) process.^16^ For this reason, the JAEA has proposed a so-called “milking” technique^17^ in which high-density α-MoO_3_ microspheres are irradiated and dissolved in a NaOH solution. Following this, ^99^Mo is absorbed using PZC (KAKEN Co, PZC-BE22) after which the ^99m^Tc solution is concentrated using 2-butanone.^18^ However, this method is subject to numerous limitations. Firstly, the high-density α-MoO_3_ pellets are not readily sintered and are easily broken during the dissolution process.^19^ Secondly, this technique generates a large amount of radioactive waste liquid that is subsequently difficult to handle.^17^ Thirdly, it remains necessary to devise a means of separating the ^99^Mo isotope from ^98^Mo. Therefore, a novel extraction technique that is compatible with various types of irradiation targets and minimizes radioactive contamination is required.

Hot atom chemistry (HAC), referring to the chemical reactions that occur between high-energy atoms, ions and molecules,^20^ was first described by Szilard and Chalmers in 1934.^21^ In early experiments, ethyl iodine was irradiated with neutrons, leading to a ^127^I (n, γ) ^128^I nuclear reaction, after which much of the resulting ^128^I could be extracted with water,^21^ showing that the ^128^I had separated from the parent compound. This phenomenon could potentially be applied to the manufacture of radioisotopes with medical applications. In fact, a technique for producing ^99m^Tc using HAC was proposed in 2019.^22^ In this process, a MoO_3_ target is neutron-irradiated to promote the ^98^Mo (n, γ) ^99^Mo nuclear reaction, followed by extraction of the ^99^Mo using water. The lower solubility of MoO_3_ in water ensures the purity of the resulting solution. The efficacy of this treatment was demonstrated by Ngo *et al.* and Quach *et al.* in studies using β-MoO_3_ and α-MoO_3_, respectively.^23,24^ Even so, the authors have encountered challenges in terms of synthesizing and recycling irradiation targets such as α-MoO_3_ pellets and β-MoO_3_ whiskers. Therefore, it would be helpful to devise a new extraction method that is compatible with various types of irradiation targets.

Large amounts of energy are released in conjunction with nuclear reactions, a small part of which can be passed on as recoil energy to isotope atoms such that these atoms separate from the molecules in which they are bound.^25^ It stands to reason that this process will be affected by both elapsed time and temperature. If the separation process can be accelerated by increasing the temperature or the length of time that the water is heated, a greater quantity of isotopes atoms could possibly be extracted. Molybdenum trioxide (MoO_3_) has been used as a neutron-irradiation target.^15^ This compound has also been employed in catalyst systems, gas sensors, electronics and optical materials.^26,27^ MoO_3_ has three different crystal forms: orthorhombic α-MoO_3_, monoclinic β-MoO_3_ and triclinic h-MoO_3_,^28^ and α-MoO_3_ pellets and β-MoO_3_ whiskers have been used to produce ^99m^Tc.^18,23^ However, as noted, the synthesis and recycling of these materials has been found to be difficult.^19,29^ For this reason, β-MoO_3_ particles were selected as the irradiation target for ^99m^Tc production in the present work.

In the present research, firstly, β-MoO_3_ particles were prepared through thermal evaporation of α-MoO_3_ powders in a tube furnace under flowing O_2_ gas. Then the β-MoO_3_ particles were characterized using X-ray diffraction (XRD) and transmission electron microscopy (TEM). Next, the β-MoO_3_ particles were irradiated with neutrons at the Kyoto University Research Reactor (KUR). A small quantity of the irradiated particles was analyzed by a high-purity germanium semiconductor detector (HPGe). The remaining β-MoO_3_ particles were divided into portions, each of which was thoroughly mixed with water at temperatures of 20 to 50 °C. After that, the configured solutions were subjected to heating 1–5.5 hours. β-MoO_3_ solution samples were prepared at various temperatures and heating times and also analyzed by HPGe. The concentrations of ^98^Mo in the solutions were determined using an inductively coupled plasma mass spectrometer (ICP-MS). From these data, temperature and time dependence of ^99^Mo extraction efficiency was confirmed. Possible extraction process is discussed.

## 2. Experimental

### 2.1 Preparation of targets

Irradiation targets of β-MoO_3_ particles were prepared through thermal evaporation of α-MoO_3_ powders in a tube furnace.^29^ The α-MoO_3_ powder, as a raw material, was heated and vaporized at a temperature of 900 °C. The Mo–O vapor was cooled in flowing O_2_ gas at 60 kPa to form β-MoO_3_ particles. β-MoO_3_ particles were examined using a transmission electron microscope (TEM, HT7700) operated at an acceleration voltage of 100 kV. The phase was determined through X-ray diffraction (XRD, MiniFlex600) analysis using Cu-Kα radiation with a wavelength of 0.1518 nm.

### 2.2 Irradiation of targets

A 0.4 g mass of β-MoO_3_ particles was securely sealed placed inside a polyethylene capsule as the target for neutron irradiation. The sample was exposed to a neutron flux (*Φ*) of 3 × 10^13^ neutrons·cm^−2^·s^−1^ for a duration of 20 min using a Pn-2 apparatus operating at a power level of 5 MW at the KUR facility. The irradiated samples were subsequently allowed to stand undisturbed for four days to allow the natural decay of radioactivity prior to the extraction trials.

### 2.3 Extraction of ^99^Mo into solution

**Fig. 1:**
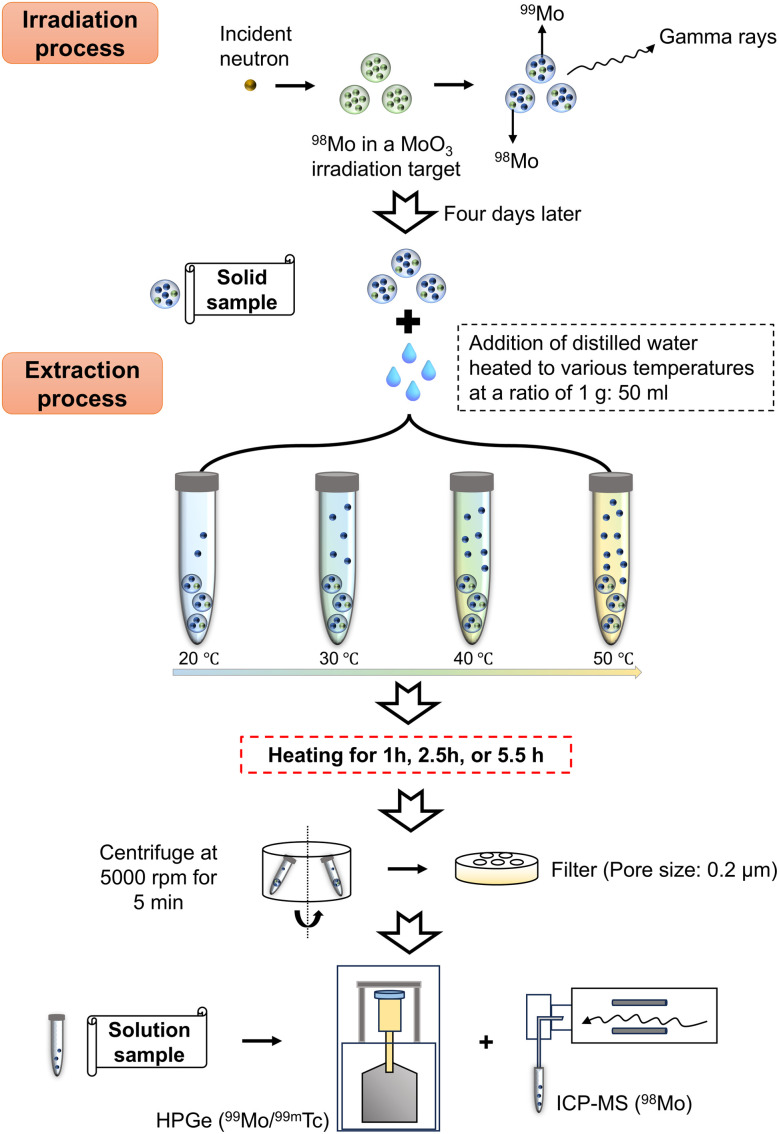
Experimental process.

The process of removing the irradiated samples from the capsules inevitably produced a slight reduction in mass. Small portions of the irradiated β-MoO_3_ particles (that is, the solid samples) were taken from the irradiation capsules, weighed and prepared for the acquisition of γ-ray spectra using a high-purity germanium semiconductor detector (HPGe, Mirion Technologies Canberra, GC4020, FWHM: 1.4 keV at 140.5 keV) to determine the activity of the ^99^Mo. The measurement time employed to assess a solid sample is defined herein as *t* = *t*_1_. The remaining irradiated β-MoO_3_ particles were divided into four portions, each of which was weighed and labeled individually. The distilled water at temperatures of 20, 30, 40, and 50 °C were added into the irradiated β-MoO_3_ particles at a ratio of 1 g/50 ml at *t* = *t*_0_. After thorough mixing of these particles with the water, β-MoO_3_ dispersed solutions were kept at temperatures of 20, 30, 40, and 50 °C for total heating times of 1, 2.5, and 5.5 hours using a hot stirrer. Following this, each sample was subjected to centrifugation at 5000 rpm for 5 min and a needle filter (PTFE013045) with a pore size of 0.2 μm was utilized to extract 0.05 ml of each solution and to then deposit this aliquot onto a small paper disc (referred to herein as a solution sample). The solution sample also analyzed by HPGe and the measurement time is defined herein as *t* = *t*_2_. After one month, the concentration of ^98^Mo in the solutions was ascertained by ICP-MS (Plasma Quant MS Elite). Notes: to ensure experimental accuracy, two replicates were prepared for each solid and solution sample. During the analyses of specimens using the HPGe detector, a BE8302 standard was employed to calibrate the energy and activity for both the solid and solution samples. Using the masses of these specimens and the half-life of ^99^Mo (66.59 h), the activities of the solid samples at the time of the solution sample activity measurements (*t* = *t*_2_) were calculated. The extraction efficiency of ^99^Mo was defined as the ratio of the ^99^Mo activity in the solution sample to that in the solid sample at *t* = *t*_2_. The mass of ^98^Mo in each specimen was calculated based on the concentration, and the extent of extraction of ^98^Mo was defined as the mass of ^98^Mo in the solution divided by the mass of ^98^Mo in the solid. Fig. 1 presents a flowchart summarizing the experimental procedures.

## 3. Results

### 3.1 Morphology and phase composition of targets

**Fig. 2:**
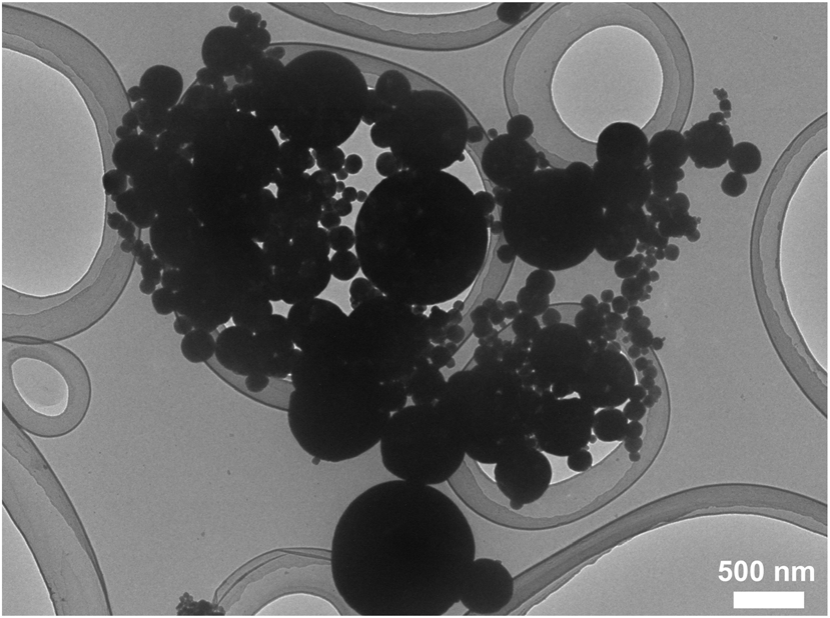
Bright-field TEM image of synthesized β-MoO_3_ particles.

**Fig. 3:**
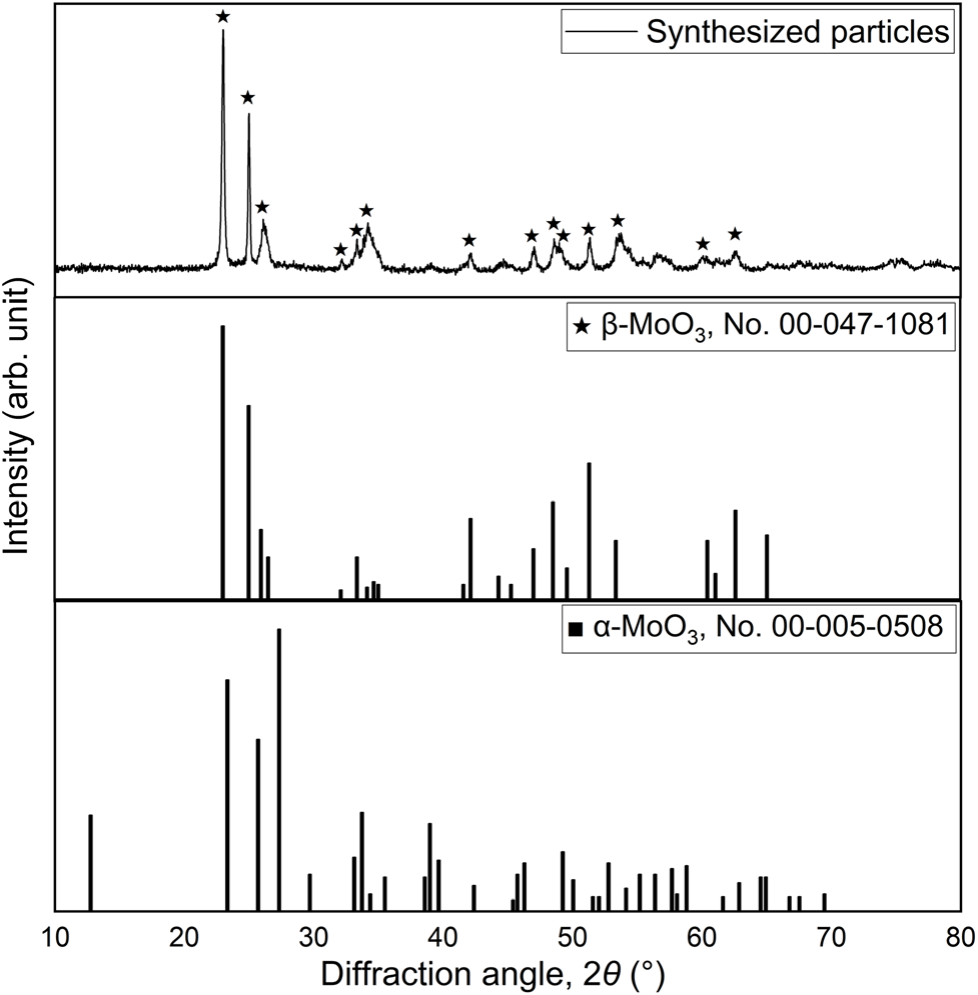
XRD patterns for synthesized β-MoO_3_ particles and for reference materials. The reference diffraction peaks are taken from the ICDD Powder Diffraction File (PDF2.DAT) database.”

A bright-field TEM image of β-MoO_3_ particles synthesized is shown in Fig. 2. This image displays spherical particles with sizes between 50 and 1000 nm. This material was determined to comprise a single β-MoO_3_ phase based on the XRD data provided in Fig. 3. From these results, it was concluded that spherical β-MoO_3_ particles were successfully synthesized by the thermal evaporation method.

### 3.2 Gamma-ray spectrum

**Fig. 4:**
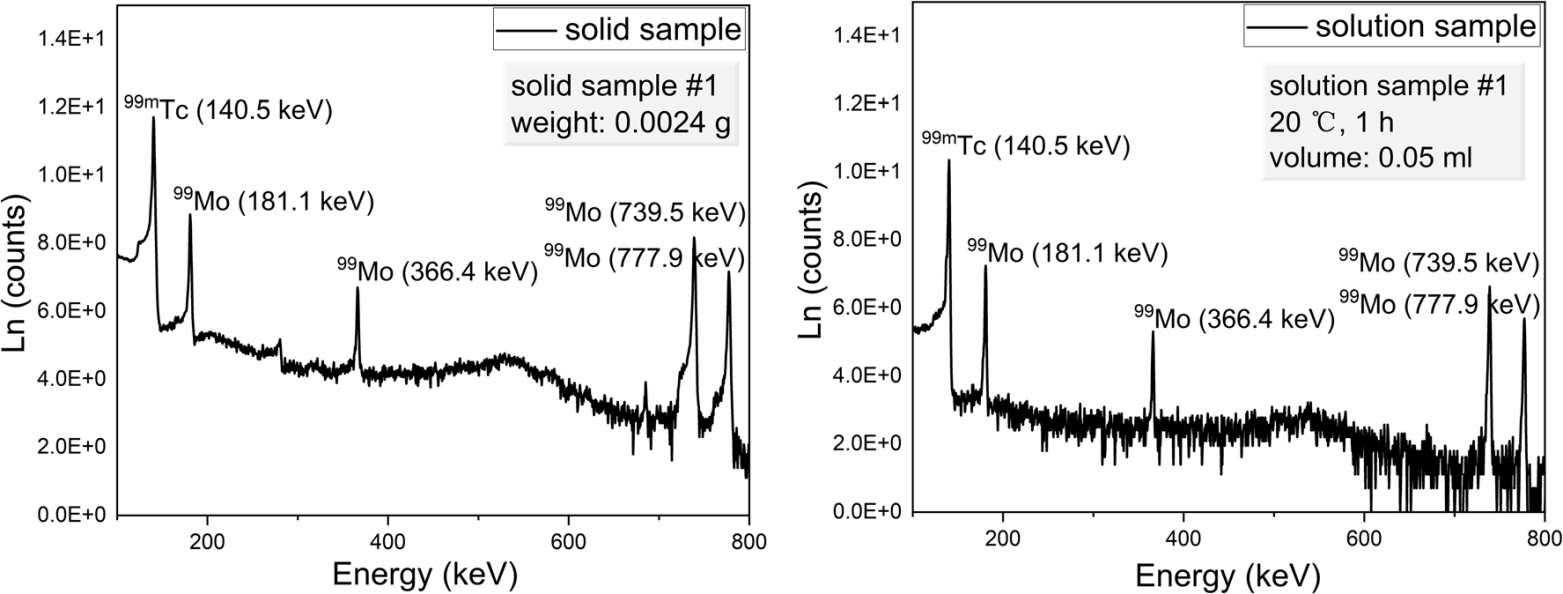
The gamma ray spectra of representative solid and solution samples.

**Fig. 5:**
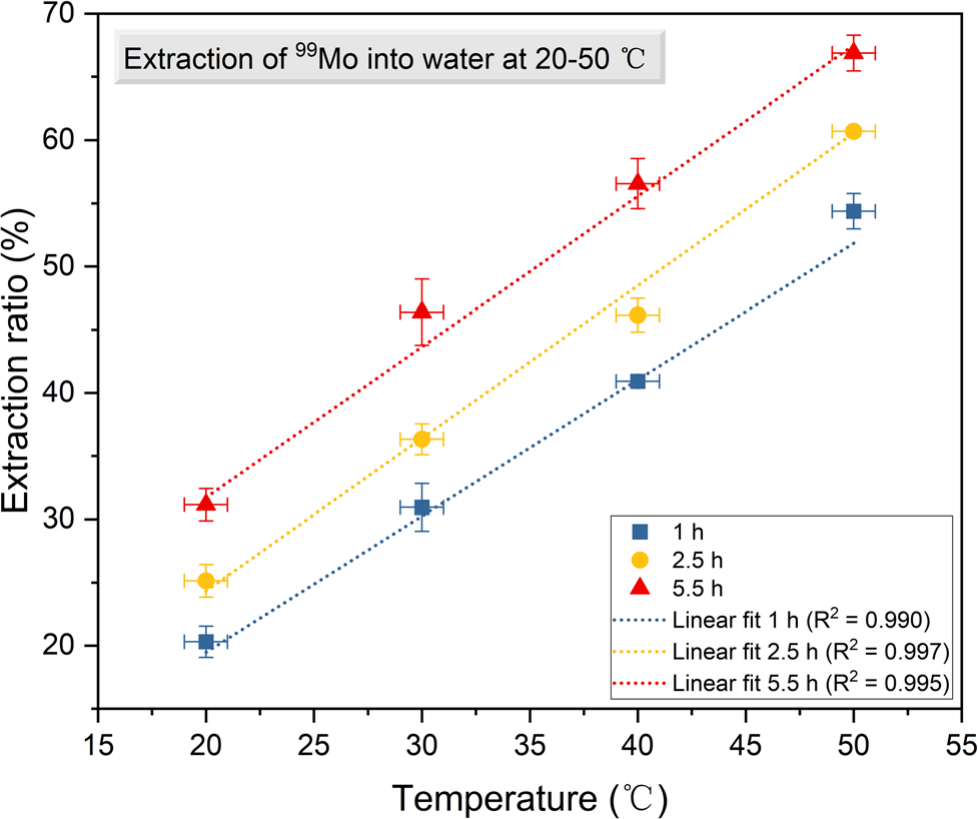
The extraction efficiency of ^99^Mo as a function of temperature for various heating durations.

**Fig. 6:**
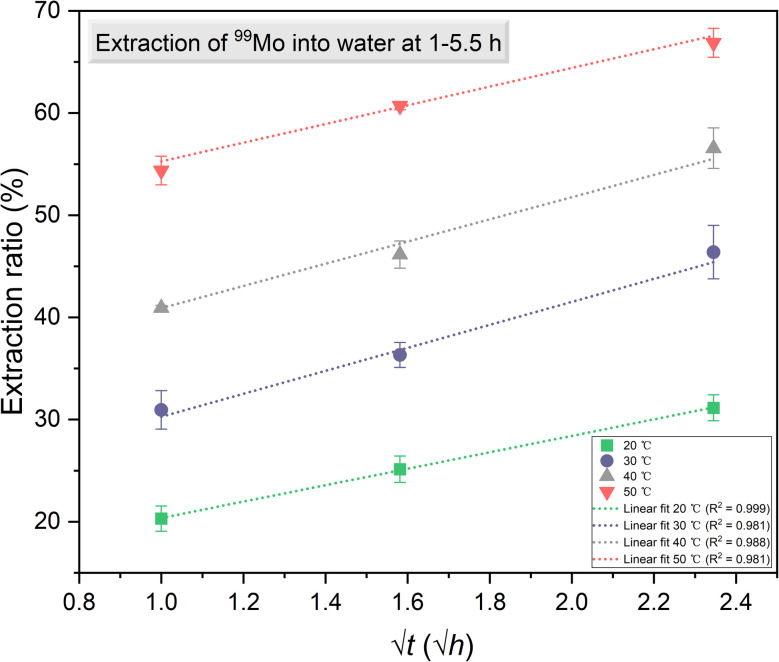
The extraction efficiency of ^99^Mo as a function of heating time for various temperatures.

The gamma ray spectra acquired from solid and solution samples are shown in Fig. 4. These spectra exhibit distinct peaks corresponding to ^99m^Tc and ^99^Mo at energies of 140.51, 181.1, 366.4, 739.50 and 777.9 keV. The data obtained using the HPGe detector are summarized in Tables 1 and 2 while the details of the associated calculations can be found in the ESI.† Note that these calculations were based on procedures previously reported in the literature.^30,31^ As noted, a BE8302 standard was used for the energy and activity calibrations and the activities of both the solid and solution specimens were calculated at *t* = *t*_2_ to determine the extraction efficiencies. These values were calculated by dividing the activities of the solutions by those of the solids. Plots of the extraction efficiencies *versus* temperature for various heating durations and *versus* time for various temperatures are provided in Fig. 5 and 6, respectively. For clarity and better visualization, all linear regression equations used for curve fitting are presented in Table 5 at the end of the manuscript, while only *R*^2^ values are shown in the corresponding figures. These data demonstrate that the extraction efficiency increased with increases in both temperature and heating duration. By varying the time and temperature, the extraction efficiency could be increased from 20.31 ± 1.24% to 66.88 ± 1.42%.

**Table 1:** The effect of temperature on ^99^Mo extraction for a duration of 5 h

Temperature (°C)	Time (h)	Sample number	Solid samples		Solution samples		Extraction efficiency (%)
			Measured intensity (counts)	Total activity at *t* = *t*_2_ (Bq)	Measured intensity (counts)	Total activity at *t* = *t*_2_ (Bq)	
20	5.5	#1	11 090	0.385	2557	0.115	29.88
		#2	12 515	0.359	2591	0.116	32.42
30		#1	11 090	0.392	3728	0.171	43.76
		#2	12 515	0.366	3898	0.179	49.00
40		#1	11 090	0.448	4627	0.244	54.58
		#2	12 515	0.418	4635	0.245	58.54
50		#1	11 090	0.303	4924	0.198	65.46
		#2	12 515	0.283	4798	0.193	68.30

**Table 2:** The effect of heating time on ^99^Mo extraction for a temperature of 50 °C

Temperature (°C)	Time (h)	Sample number	Solid samples		Solution samples		Extraction efficiency (%)
			Measured intensity (counts)	Total activity at *t* = *t*_2_ (Bq)	Measured intensity (counts)	Total activity at *t* = *t*_2_ (Bq)	
50	1	#1	11 090	0.315	4142	0.167	52.97
		#2	12 515	0.294	4073	0.164	55.77
	2.5	#1	11 090	0.309	4626	0.186	60.32
		#2	12 515	0.288	4374	0.176	61.07
	5.5	#1	11 090	0.303	4924	0.198	65.46
		#2	12 515	0.283	4798	0.193	68.30

### 3.3 ICP-MS result

**Fig. 7:**
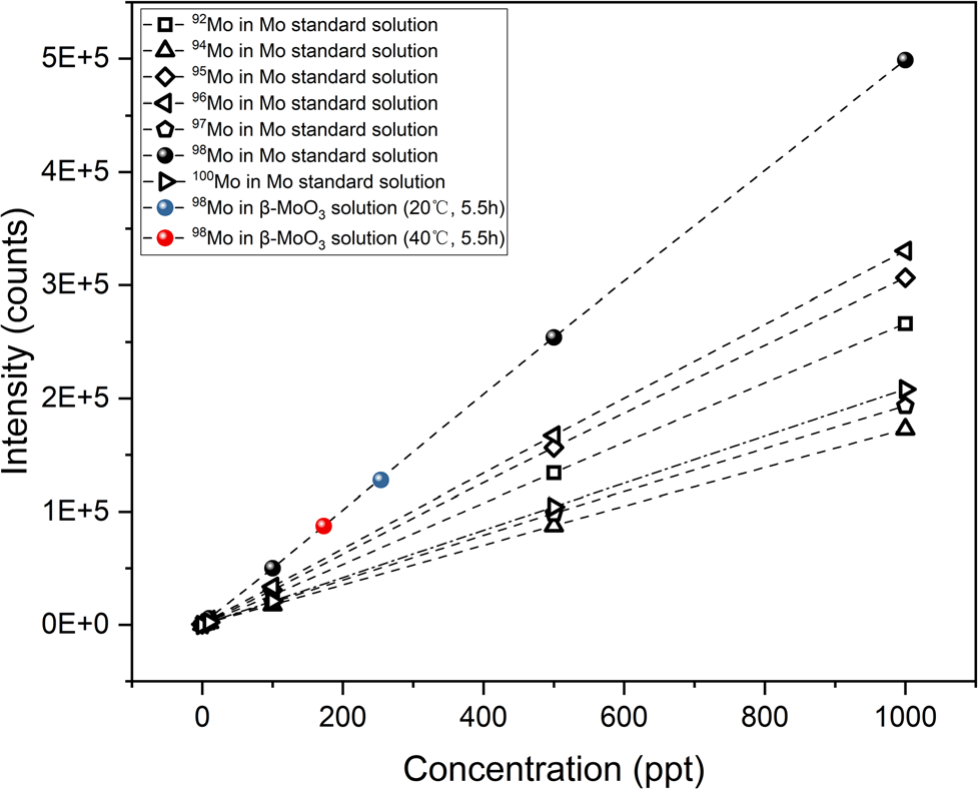
The concentrations of ^98^Mo in Mo standard solutions and β-MoO_3_ solutions at 20 and 40 °C.

MoO_3_ is insoluble in water, and the natural abundance of ^98^Mo is 24.39%, being higher than that of other isotopes.^32^ Hence, the ^98^Mo and ^99^Mo extraction efficiencies are compared herein. After one month, the concentration of ^98^Mo in the solutions was determined by ICP-MS and ^98^Mo extraction efficiency was defined as the mass of ^98^Mo in the solution divided by the initial mass of ^98^Mo in the solid. The results of these trials are provided in Fig. 7 and Table 3 while the process used for the association calculations can be found in the ESI.† In order to ensure the reliability of isotope quantification by ICP-MS, multiple Mo isotopes were monitored simultaneously. Fig. 7 shows the signal consistency across isotopes, which confirms the linearity and robustness of the measurement system. The results confirmed that the ^98^Mo extraction efficiencies in the solutions heated to 20 and 40 °C were much lower than the values obtained for ^99^Mo, indicating the efficacy of the hot atom mechanism at varying temperatures. The amounts of ^99^Mo (derived from the activity data) and of ^98^Mo (based on concentration) were also calculated, as shown in Table 3. However, it should be noted that the ratio of ^99^Mo to ^98^Mo is extremely low. This is primarily due to the short half-life of ^99^Mo, which leads to high specific activity. As a result, even a minimal mass can generate substantial radioactivity, and radioactivity is the primary factor determining the material's usefulness in medical applications.

**Table 3:** Concentrations of ^98^Mo in solutions following heating at two different temperatures

Samples	Counts	Concentration of ^98^Mo from ICP-MS (ppt)	Extraction efficiency of ^98^Mo (%)	Calculated ^99^Mo/^98^Mo Mass ratio
β-MoO_3_, 20 °C	127 831	2.54 × 10^9^	4.48	5.38 × 10^−16^
β-MoO_3_, 40 °C	87 315	1.73 × 10^9^	3.05	1.42 × 10^−15^

## 4. Discussion

### 4.1 Hot atoms at high temperature

The values for ^99^Mo extraction efficiency from irradiated β-MoO_3_ particles, as summarized in Fig. 5, are similar to the efficiencies of extraction from β-MoO_3_ whiskers in previous research.^23^ The present results therefore reconfirm the advantage of using β-MoO_3_ compared with α-MoO_3._ As noted, hot atoms having high kinetic or internal energies are generated during nuclear reactions^33^ and the recoil energy transferred to newly formed radioactive atoms during this process can rupture chemical bonds.^9–13^ In previous work by the authors, the neutron irradiation reaction ^98^Mo (n, γ) ^99^Mo generated ^99^Mo atoms possessing a kinetic energy of 193 eV.^23^ These atoms could be extracted from the solid phase into solution, and decay into ^99m^Tc. It is also apparent from Fig. 5 and 6 that increasing the temperature of the water used for extraction and prolonging the duration of extraction increased the efficiency of this process. Evidently, increasing the water temperature speeds up the movement of hot atoms.

### 4.2 Extraction process of ^99^Mo into water

To investigate the extraction of ^99^Mo hot atoms into water in more detail, the reaction order was estimated based on the rate law. This law is commonly used to describe the relationship between the rate of a chemical reaction and the concentration of reactants, such that reactions are categorized as either zero, first or second order or complex processes.^34^ To determine the reaction order, the concentration of Mo-99 as a function of time was derived from the radioactivity measured by a germanium detector. The data were fitted to zero-order, first-order, and second-order kinetic models, and the corresponding coefficients of determination (*R*^2^) and residual sum of squares (RSS) were calculated. The results are summarized in Fig. S4 and Table S5† of the ESI.† Comparative analysis indicates that the first-order kinetic model with an offset term provides the best overall fit across different temperature conditions. The presence of the offset term in the first-order model suggests the existence of a “release limit” within the system. Such limiting behavior has been widely reported in systems involving porous materials or diffusion-restricted matrices.^35,36^ This result indicates that the process being modeled can be summarized simply asReactant (*X*)→Product (*Y*).

Based on the ^99^Mo activities in the solids and solutions (Table 1), the ^99^Mo concentrations were calculated from the equation^37^1
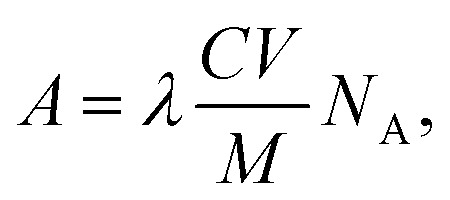
where *A* is the activity, *λ* is the decay constant, *C* is the concentration, *V* is the volume of solution, *N*_A_ is Avogadro's number and *M* is the molar mass of the radioactive nuclide. The integral rate equation^38^ used in this work was

**Fig. 8:**
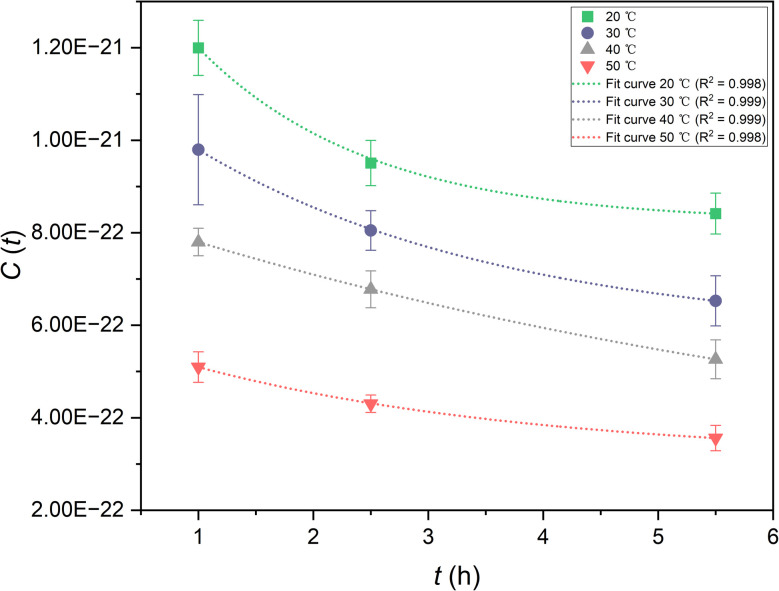
The ^99^Mo concentrations in the solid specimens as functions of time.

2 *C*_*t*_ = *C*_0_e^−*kt*^,

where *C*(*t*) is the concentration of the reactant at time *t*, *C*_o_ is the initial concentration of the reactant, *k* is the rate constant and *t* is the time. The relationships between the concentration of ^99^Mo and time for various temperatures are shown in Fig. 8. After fitting the experimental data, the fitting function was found to contain an offset term (*y*_0_), indicating that the system had reached an equilibrium state such that the concentration approached *y*_0_ rather than going to zero. The integral rate equation was subsequently substituted into the equation defining the efficiency of ^99^Mo extraction into water, *E*, to give3
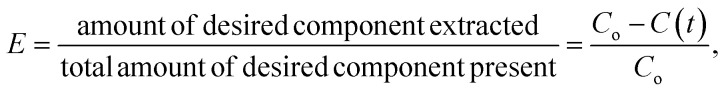
where *E* denotes the efficiency of Mo-99 transfer from solid to aqueous phase. Finally, the rate constant, *k*, was calculated from eqn (2) and (3) as4
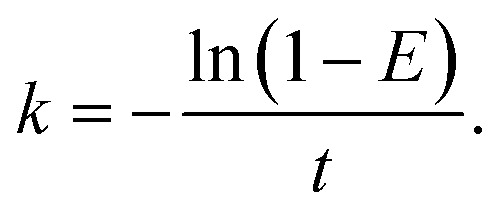


The rate constants at different temperatures were subsequently calculated using eqn (4) in conjunction with the extraction efficiency data. These values were used to determine the activation energy for the reaction. Activation energy is a crucial parameter in kinetic studies and can be employed to infer the reaction mechanism. In the Arrhenius reaction rate model, activation energy is defined as the minimum energy required for reactants to undergo a chemical reaction.^39^ The Arrhenius equation provides the correlation between activation energy and reaction rate,^40^ written as

5 *k* = *B*e−^*E*_a_/(*RT*)^,

where *B* represents the pre-exponential factor for the reaction, *E*_a_ is the activation energy, *R* is the universal gas constant and *T* is the absolute temperature. This equation can be transformed into a correlation between ln(*k*) and 1/*T* after a natural logarithm conversion, giving6
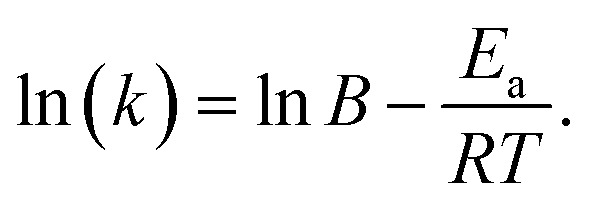


**Fig. 9:**
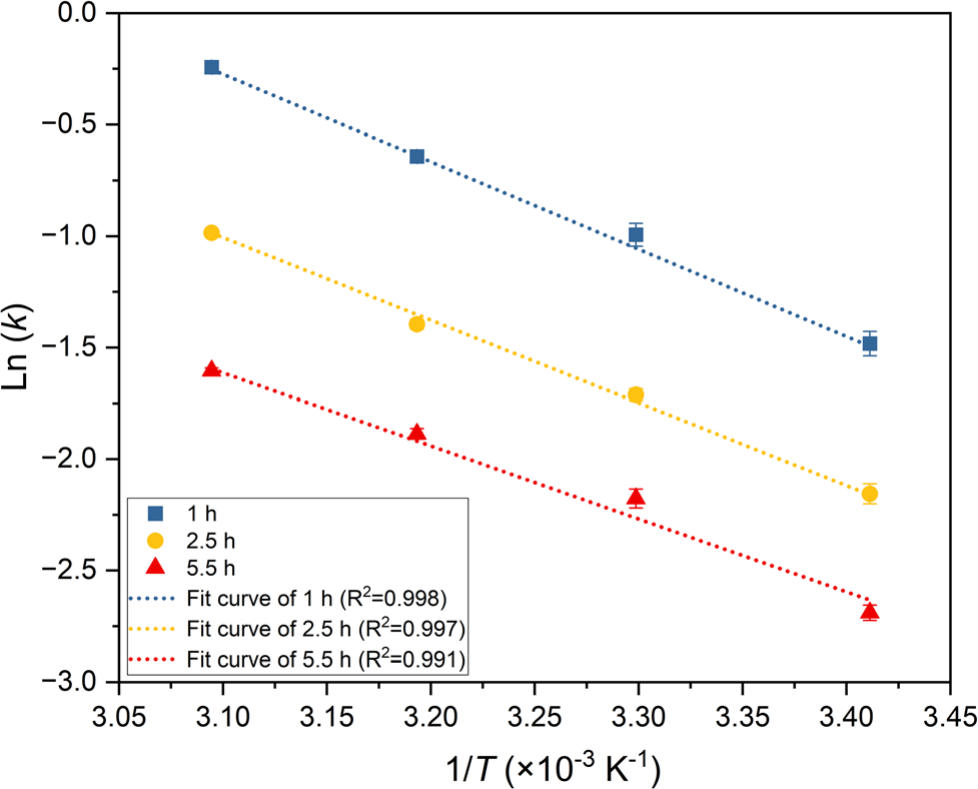
Plot of ln(*k*) against 1/*T* for various extraction times.

Hence, a plot of ln(*k*) as a function of 1/*T*, as depicted in Fig. 9, can be used to find *E*_a_. The values determined for the extraction of ^99^Mo hot atoms from the target into water are presented in Table 4.

**Table 4:** Activation energy for the extraction of ^99^Mo hot atoms from irradiated β-MoO_3_ into water

Samples	Activation energy (kJ mol^−1)^
β-MoO_3_, 1 h	32.60
β-MoO_3_, 2.5 h	30.85
β-MoO_3_, 5.5 h	27.25

**Table 5:** Summary of linear regression equations corresponding to Fig. 5–9. *R*^2^ values indicate the goodness of fit for each regression

Figure	Curve label	Fitting type	Equation	*R* ^2^ value
Fig. 5	1 h	Linear	*y* = 1.079*x* − 2.116	0.990
	2.5 h	Linear	*y* = 1.208*x* + 0.173	0.997
	5.5 h	Linear	*y* = 1.192*x* + 7.864	0.995
Fig. 6	20	Linear	*y* = 8.049*x* + 12.309	0.999
	30	Linear	*y* = 11.249*x* + 19.027	0.981
	40	Linear	*y* = 10.866*x* + 30.040	0.988
	50	Linear	*y* = 9.148*x* + 46.121	0.981
Fig. 7	^98^Mo	Linear	*y* = 499.353*x* + 972.903	0.999
Fig. 8	20	Exponential	*y* = 7.389 e^−0.674*x*^ + 8.232	0.999
	30	Exponential	*y* = 5.835 e^−0.370*x*^ + 5.763	0.999
	40	Exponential	*y* = 6.388 e^−0.135*x*^ + 2.214	0.999
	50	Exponential	*y* = 2.754 e^−0.339*x*^ + 3.133	0.999
Fig. 9	1 h	Linear	*y* = −3.920*x* + 11.878	0.998
	2.5 h	Linear	*y* = −3.710*x* + 10.498	0.997
	5.5 h	Linear	*y* = −3.277 *x* + 8.545	0.991

These activation energies are in the range of 27.25–32.60 kJ·mol^−1^ and so are lower than those reported for the leaching of Mo from MoO_3_ into NH_3_ (44.53 kJ·mol^−1^) or the dissolution of MoO_3_ in KOH (47.81 kJ·mol^−1^),^41,42^ suggesting that the present experiments were not simple dissolution of MoO_3_ in water. Furthermore, the reported energy values for the migration of oxygen deficiencies from MoO_3_ to MoO_2_ (122 kJ·mol^−1^) and for Mg diffusion in MoO_3_ (47 kJ·mol^−1^)^43,44^ are higher than the present results, and so the diffusion or reaction of anions and cations the anion/cation diffusion/reaction are not simple rate controlling process. Interestingly, the activation energies for the hydration of MoO_3_ and for proton conductivity in H_*x*_MoO_3_ (9–28 kJ·mol^−1^)^45^ are lower than or comparable to the present results. It is known that β-MoO_3_ can only form a hydrated phase in water at low temperatures,^46^ which must take place in the present extraction process. Therefore, from the calculated activation energies of 27.25 to 32.60 kJ mol^−1^, it is apparent that the extraction process may involve the diffusion of atoms through a hydrated phase. Further experimental work is needed to precisely determine the reaction mechanism governing this process.

## 5. Conclusions

This study investigated the extraction behavior of ^99^Mo hot atoms from neutron-irradiated β-MoO_3_ particles into water under varying temperature and time conditions. The extraction efficiency was found to increase significantly with both rising temperature and prolonged heating, improving from 20.31 ± 1.24% to 66.88 ± 1.42%. Kinetic analysis indicated that the process follows a first-order reaction model, and the calculated activation energy ranged from 27.25 to 32.60 kJ mol^−1^, suggesting the diffusion of atoms through a hydrated phase. These findings demonstrate the critical role of thermal treatment in enhancing hot atom release from solid targets and provide valuable insights for optimizing the post-irradiation handling of molybdenum-based materials used in medical radioisotope production.

## Data availability

The data supporting this article have been included as part of the ESI.†

## Conflicts of interest

There are no conflicts of interest to declare.

## Supplementary Material

RA-015-D5RA01952D-s001
