# Supplementary material for: Temperature and time dependence on extraction of Molybdenum-99 hot atoms from neutron-irradiated β-molybdenum trioxide particles into water
Source: RSC Adv. 2025 May 22;15(22):17222–9. doi: 10.1039/d5ra01952d (PMC12096280; doi:10.1039/d5ra01952d)
Supplement: RA-015-D5RA01952D-s001 [file RA-015-D5RA01952D-s001.pdf]

Supplementary Information (SI) for RSC Advances.
This journal is © The Royal Society of Chemistry 2025

Supplementary material

Activity calculation

The procedure for determining sample activity aligns with the methodology outlined in Refs.[30,31] Initially, A standard radioactive source, STD-BE8302, shown in Fig. S1, was detected by a germanium detector to obtain its gamma-ray energy spectrum. The number of photons detected by a germanium detector is always lower than the number of photons emitted by the radioactive source, and the detection efficiency represents the ratio between them,

$$\varepsilon = \frac{N_{meas}}{N_{emit}} \quad (1)$$

where, N_{meas} is the number of counts (photons) observed by the detector, N_{emit} is the number of photons emitted by the source. N_{meas} is the net area of the full absorption peak at each energy spectral line. N_{emit} is derived by multiplying the rate of gamma-ray emission with the duration of measurement. Therefore, the detection efficiency of individual nuclides can be calculated. The available data regarding the standard samples is presented in Tables S1 and S2. The relationship between energy and detection efficiency is illustrated in Figs. S2 and S3. The gamma ray energies of ^{99}Mo and ^{99m}Tc are 739.5keV and 140.51keV, respectively, while the corresponding detection efficiencies can be determined. β-MoO_3 solid and solution samples were detected as radioactive sources in the germanium detector, respectively. N_{emit} can be determined from N_{meas} and ε. The numbers of photons emitted by the radioactive source can be determined the following equation[19)],

$$N_{emit} = AYt \quad (2)$$

where, N_{emit} is the number of photons emitted by the source, A is the activity in disintegrations per second. Y is the gamma-ray yield. t is the time in seconds.

The gamma ray yields for ^{99}Mo and ^{99m}Tc are 12.5% and 87.5%, respectively. Therefore, the activity of the solid and solution samples can be calculated. The activity of solid samples was presented in Table S3. The activity of solution samples was shown in Table S4. The activity extraction ratio was obtained by dividing the activity of the solution sample by the activity of the solid sample. Notes: The standard sample were positioned at location 5, where the solid sample was placed, and also at location 1, where the solution sample was placed. The activity of solid and solution samples was calculated at the decay time $t = t_2$ for calculating extraction ratio. The total weights of solid and solution samples used to calculate final activity remain constant.

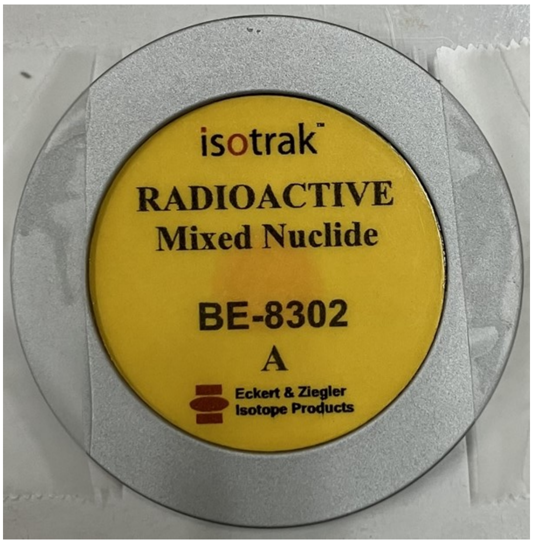

Figure S1. Standard sample of BE8302.

Table S1. Standard sample data at position 5.

STD-BE8302-position 5	measure counts	measure time (s)	emission rate (s^{-1})	emission rate at $t=t_1$ (s^{-1})	emit counts	efficiency
Am-241	76433	3600	1220	1217	4381952	0.01744
Cd-109	29415	3600	589	265	955432	0.03079
Co-57	15511	3600	504	133	478630	0.03241
Ce-139	4580	3600	661	47	170494	0.02686
Tin-113	4105	3600	1820	79	283515	0.01448
Cs-137	72712	3600	2210	2139	7699322	0.00944
Yt-88	4743	3600	5720	175	630712	0.00752
Co-60	49248	3600	3090	2561	9219895	0.00534
Co-60	48841	3600	3100	2569	9249733	0.00528
Yt-88	2872	3600	6060	186	668202	0.00430

Table S2. Standard sample data at position 1.

STD-BE8302-position 1	measure counts	measure time (s)	emission rate (s^{-1})	emission rate at $t=t_2$ (s^{-1})	emit counts	efficiency
Am-241	175881	3600	1220	1217	4381952	0.04014
Cd-109	84700	3600	589	265	955432	0.08865
Co-57	48935	3600	504	133	478630	0.10224
Ce-139	15853	3600	661	47	170494	0.09298
Tin-113	13240	3600	1820	79	283515	0.04670

Cs-137	231218	3600	2210	2139	7699322	0.03003
Yt-88	13403	3600	5720	175	630712	0.02125
Co-60	148042	3600	3090	2561	9219895	0.01606
Co-60	129775	3600	3100	2569	9249733	0.01403
Yt-88	7566	3600	6060	186	668202	0.01132

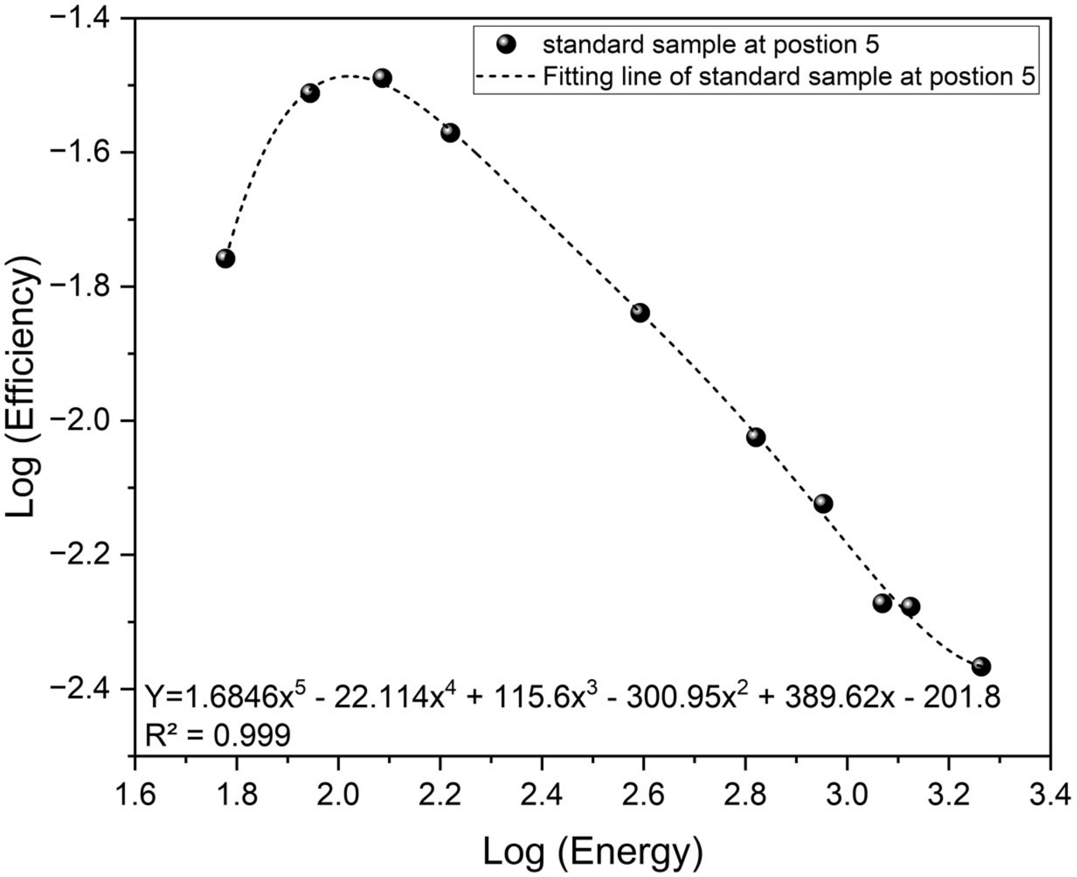

Figure S2. The relationship between energy and detection efficiency of standard sample at position 5.

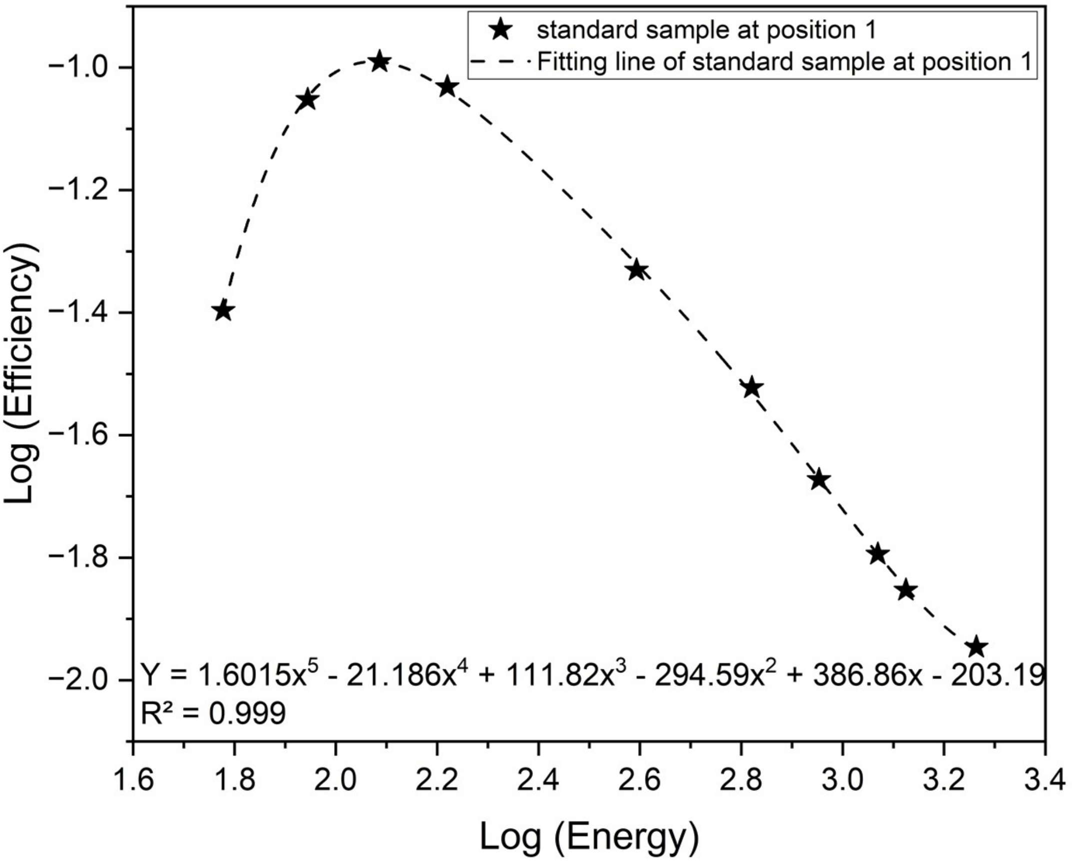

Figure S3. The relationship between energy and detection efficiency of standard sample at position 1.

Table S3. The activity of solid samples.

solid-position 5	measure counts	efficiency	emit counts	measure time (s)	branching percentage	activity (MBq)	measure weight (g)	total weight (g)	total activity at t=t_1 (MBq)	t_1-t_2 (h)	total activity at t=t_2 (MBq)
β-20℃-1h-1	11090	0.009007	1231201	600	0.125	0.016	0.0024	0.0954	0.653	30.38	0.474
β-20℃-1h-2	12515	0.009007	1389404	600	0.125	0.019	0.0029	0.0954	0.609	30.38	0.443
β-30℃-1h-1	11090	0.009007	1231201	600	0.125	0.016	0.0024	0.0976	0.668	30.85	0.483
β-30℃-1h-2	12515	0.009007	1389404	600	0.125	0.019	0.0029	0.0976	0.623	42.37	0.399
β-40℃-1h-1	11090	0.009007	1231201	600	0.125	0.016	0.0024	0.1121	0.767	42.83	0.489
β-40℃-1h-2	12515	0.009007	1389404	600	0.125	0.019	0.0029	0.1121	0.716	42.83	0.456
β-50℃-1h-	11090	0.009007	1231201	600	0.125	0.016	0.0024	0.0854	0.584	58.82	0.315

1											
β-50℃-1h-2	12515	0.009007	1389404	600	0.125	0.019	0.0029	0.0854	0.546	58.82	0.294
β-20℃-2.5h-1	11090	0.009007	1231201	600	0.125	0.016	0.0024	0.0954	0.653	46.57	0.400
β-20℃-2.5h-2	12515	0.009007	1389404	600	0.125	0.019	0.0029	0.0954	0.609	46.57	0.373
β-30℃-2.5h-1	11090	0.009007	1231201	600	0.125	0.016	0.0024	0.0976	0.668	47.02	0.407
β-30℃-2.5h-2	12515	0.009007	1389404	600	0.125	0.019	0.0029	0.0976	0.623	47.02	0.380
β-40℃-2.5h-1	11090	0.009007	1231201	600	0.125	0.016	0.0024	0.1121	0.767	47.48	0.465
β-40℃-2.5h-2	12515	0.009007	1389404	600	0.125	0.019	0.0029	0.1121	0.716	47.48	0.435
β-50℃-2.5h-1	11090	0.009007	1231201	600	0.125	0.016	0.0024	0.0854	0.584	60.67	0.309
β-50℃-2.5h-2	12515	0.009007	1389404	600	0.125	0.019	0.0029	0.0854	0.546	60.67	0.288
β-20℃-5.5h-1	11090	0.009007	1231201	600	0.125	0.016	0.0024	0.0954	0.653	50.23	0.385
β-20℃-5.5h-2	12515	0.009007	1389404	600	0.125	0.019	0.0029	0.0954	0.609	50.23	0.359
β-30℃-5.5h-1	11090	0.009007	1231201	600	0.125	0.016	0.0024	0.0976	0.668	50.68	0.392
β-30℃-5.5h-2	12515	0.009007	1389404	600	0.125	0.019	0.0029	0.0976	0.623	50.68	0.366
β-40℃-5.5h-1	11090	0.009007	1231201	600	0.125	0.016	0.0024	0.1121	0.767	51.13	0.448
β-40℃-5.5h-2	12515	0.009007	1389404	600	0.125	0.019	0.0029	0.1121	0.716	51.13	0.418
β-50℃-5.5h-1	11090	0.009007	1231201	600	0.125	0.016	0.0024	0.0854	0.584	62.5	0.303
β-50℃-5.5h-2	12515	0.009007	1389404	600	0.125	0.019	0.0029	0.0854	0.546	62.5	0.283

Table S4. The activity of solution samples.

solution-	measure	efficiency	emit	measur	branching	activity	measure	total	total activity at	extraction

position 1	counts		counts	e time (s)	percentag e	(MBq)	volume (ml)	volume (ml)	t=t_2(MBq)	ratio (%)
β-20℃-1h-1	2011	0.028297	71068	600	0.125	0.0009	0.05	4.77	0.090	19.069
β-20℃-1h-2	2122	0.028297	74991	600	0.125	0.0010	0.05	4.77	0.095	21.545
β-30℃-1h-1	3049	0.028297	107750	600	0.125	0.0014	0.05	4.88	0.140	29.055
β-30℃-1h-2	2850	0.028297	100718	600	0.125	0.0013	0.05	4.88	0.131	32.826
β-40℃-1h-1	3764	0.028297	133018	600	0.125	0.0018	0.05	5.605	0.199	40.686
β-40℃-1h-2	3557	0.028297	125703	600	0.125	0.0017	0.05	5.605	0.188	41.168
β-50℃-1h-1	4142	0.028297	146377	600	0.125	0.0020	0.05	4.27	0.167	52.972
β-50℃-1h-2	4073	0.028297	143938	600	0.125	0.0019	0.05	4.27	0.164	55.774
β-20℃-2.5h-1	2121	0.028297	74955	600	0.125	0.0010	0.05	4.77	0.095	23.846
β-20℃-2.5h-2	2195	0.028297	77570	600	0.125	0.0010	0.05	4.77	0.099	26.424
β-30℃-2.5h-1	3107	0.028297	109800	600	0.125	0.0015	0.05	4.88	0.143	35.097
β-30℃-2.5h-2	3104	0.028297	109694	600	0.125	0.0015	0.05	4.88	0.143	37.544
β-40℃-2.5h-1	3948	0.028297	139521	600	0.125	0.0019	0.05	5.605	0.209	44.813
β-40℃-2.5h-2	3906	0.028297	138036	600	0.125	0.0018	0.05	5.605	0.206	47.473
β-50℃-2.5h-1	4626	0.028297	163481	600	0.125	0.0022	0.05	4.27	0.186	60.324
β-50℃-2.5h-2	4374	0.028297	154575	600	0.125	0.0021	0.05	4.27	0.176	61.073
β-20℃-5.5h-1	2557	0.028297	90363	600	0.125	0.0012	0.05	4.77	0.115	29.876
β-20℃-5.5h-2	2591	0.028297	91565	600	0.125	0.0012	0.05	4.77	0.116	32.415
β-30℃-5.5h-1	3728	0.028297	131746	600	0.125	0.0018	0.05	4.88	0.171	43.765
β-30℃-5.5h-2	3898	0.028297	137754	600	0.125	0.0018	0.05	4.88	0.179	48.998
β-40℃-5.5h-1	4627	0.028297	163516	600	0.125	0.0022	0.05	5.605	0.244	54.576
β-40℃-5.5h-2	4635	0.028297	163799	600	0.125	0.0022	0.05	5.605	0.245	58.539
β-50℃-5.5h-1	4924	0.028297	174012	600	0.125	0.0023	0.05	4.27	0.198	65.458
β-50℃-5.5h-2	4798	0.028297	169559	600	0.125	0.0023	0.05	4.27	0.193	68.295

Comparison of Reaction Order Fits

To determine the reaction order, the concentration of Mo-99 as a function of time was derived from the radioactivity measured by a germanium detector. The data were fitted to zero-order, first-order, and second-order kinetic models, and the corresponding coefficients of determination (R^2) and residual sum of squares (RSS) were calculated. The results are summarized in Figure S4 and Table S5. Comparative analysis indicates that the first-order kinetic model with an offset term provides the best overall fit across different temperature conditions. The presence of the offset term in the first-order model suggests the existence of a "release limit" within the system. Such limiting behavior has been widely reported in systems involving porous materials or diffusion-restricted matrices.

Figure S4. Model fitting results under various reaction orders.

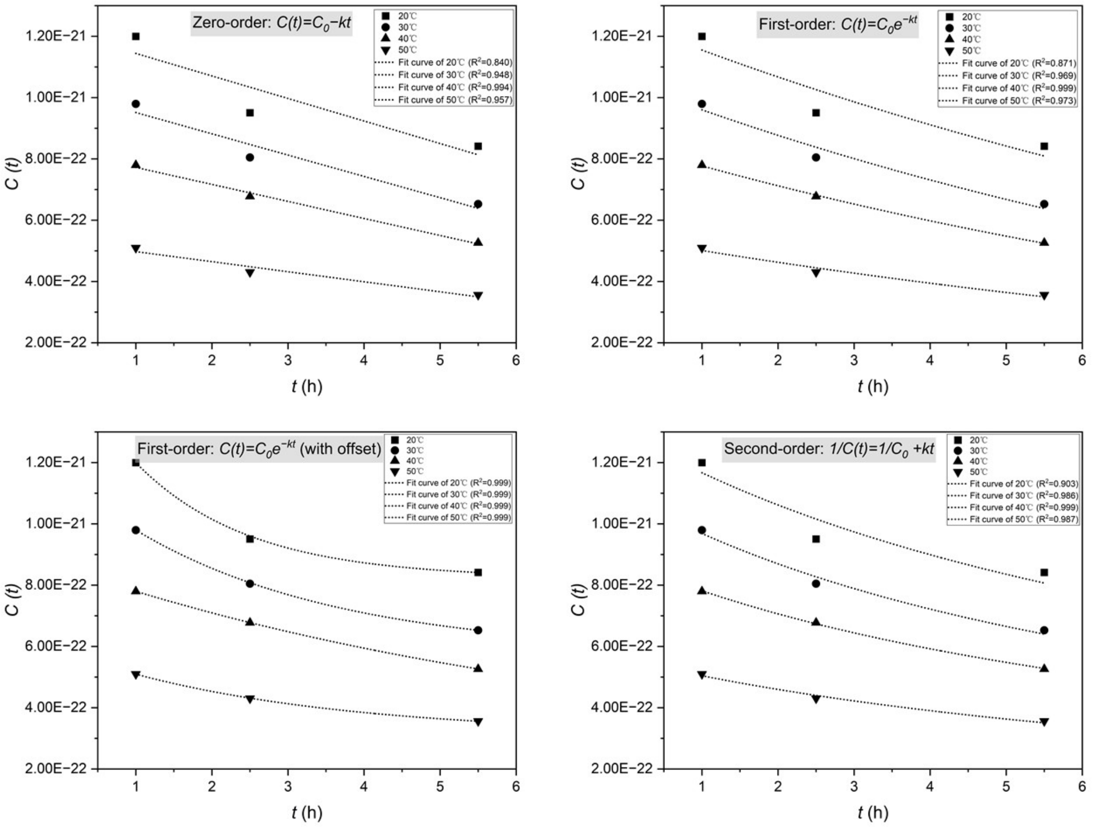

Table S5. The Comparison of Reaction Order Fits.

Temperature (℃)	Zero-order R^2	Zero-order RSS	First-order R^2 (no offset)	First-order RSS (no offset)	First-order R^2 (with offset)	First-order RSS (with offset)	Second-order R^2	Second-order RSS
20	0.84	1.08E-44	0.87	8.69E-45	0.999	0	0.90	6.51E-45
30	0.95	2.78E-45	0.97	1.68E-45	0.999	8.84E-75	0.99	7.75E-46
40	0.99	2.04E-46	0.99	2.78E-47	0.999	1.77E-74	0.99	2.02E-47
50	0.96	5.12E-46	0.97	3.17E-46	0.999	2.21E-75	0.99	1.54E-46

Concentration calculation

The concentration of ^{98}Mo was determined using an inductively coupled plasma mass spectrometry (ICP-MS) by comparing with a Mo standard solution, ICP-MS-35W-0.01X-1. The Mo standard solution with different concentrations (10ppt, 100ppt, 500ppt, 1000ppt) were prepared and subsequently quantified using the ICP-MS.

Among the isotopes of molybdenum, ^{98}Mo has the highest abundance at 24%, so calculations primarily focus on Mo-98. Given the known concentration of the standard molybdenum solution, the relationship between the count and concentration can be plotted based on the measured counts, as shown in Fig. 7. Then, β-MoO_3 solutions were analyzed using the same instrument. After measuring the counts of the β-MoO_3 solutions, according to Fig. 7, which shows the relationship between the counts and the concentration of ^{98}Mo, the concentration of ^{98}Mo of the β-MoO_3 solutions can be calculated. The calculation result is shown in Table S6.

Table S6. The calculation results from ICP-MS.

samples	counts	ppt	dilute time	final ppt	convert to g/ml	mass of ^{98}Mo in solid	mass of ^{98}Mo in solution	extraction ratio of ^{98}Mo
β-20℃	127831.227	254.047	10000000	2540469141	0.002540	0.048591667	0.00218	4.480
β-40℃	87315.289	172.910	10000000	1729095606	0.001729	0.066558333	0.00203	3.049
